# Nanotechnology-Enabled CRISPR Delivery: Emerging Opportunities in Agriculture and Forest Biotechnology

**DOI:** 10.3390/plants15142177

**Published:** 2026-07-15

**Authors:** Florin Adrian Huiban, Vladislava Galović, Maria Roberta Tripon, Saša Orlović, Camelia Tulcan, Dorin Camen

**Affiliations:** 1 Doctoral School Engineering of Plant and Animal Resources, University of Life Sciences “King Mihai I” from Timișoara, 300645 Timisoara, Romania; florin.huiban@usvt.ro (F.A.H.); roberta.tripon@usvt.ro (M.R.T.); dorincamen@usvt.ro (D.C.); 2 Research Institute for Biosecurity and Bioengineering, University of Life Sciences “King Mihai I” from Timișoara, 300645 Timisoara, Romania; 3 Institute of Lowland Forestry and Environment, University of Novi Sad, 21000 Novi Sad, Serbia; sasao@uns.ac.rs; 4 Faculty of Engineering and Applied Technologies, University of Life Sciences “King Mihai I” from Timișoara, 300645 Timisoara, Romania

**Keywords:** nanotechnology, nanoparticles, CRISPR, delivery methods

## Abstract

Genome editing is one of the key technologies in contemporary plant biotechnology, which has been revolutionized using CRISPR/Cas systems that offer rapid, flexible, and precise options to improve agricultural characteristics, enhance stress tolerance, and accelerate breeding of crops and trees. Despite the significant benefits of CRISPR/Cas systems, their use is restricted by difficulties in genome-editing materials into plant cells. The conventional approaches include Agrobacterium-mediated transformation, particle bombardment and PEG-mediated transfection; these have contributed significantly to advancements in the field; however, dependent on specific plants and requiring tissue cultures, these methods lead to random transgene insertion and poor transformation efficiency. In addition, nanotechnology represents a novel method of delivering CRISPR cargos into plant cells using minimal invasiveness and potentially without DNA. This review provides a synopsis of the most employed CRISPR/Cas systems within plants, comparing the traditional delivery mechanisms and the various nanotechnological delivery vehicles, such as lipid nanoparticles, carbon nanotubes, DNA nanostructures, mesoporous silica nanoparticles, magnetically responsive nanoparticles and green nanomaterials. This review discusses the present challenges of delivery efficacy, biocompatibility, cargo integrity, and regulatory issues, and provides suggestions for future research directions regarding nanotechnology-assisted genome editing for precision breeding, sustainable agriculture, production of crops tolerant to climate conditions, and forest biotechnology.

## 1. Introduction

The multidimensional processes driving climate change are mostly caused by anthropogenic activities, along with different threatening stressors (such as biotic and abiotic), having a negative impact on the life of crop and forest tree species, particularly the yield, growth, and physiological responses [1,2,3,4]. Advances in genome editing have shown that it is essential for protecting plants from challenging environmental conditions. When CRISPR/Cas9, a revolutionized genome-editing technology, was presented to the scientific community in 2012, this system became a powerful tool in research, providing a fast, inexpensive, and highly precise method for editing the DNA of living organisms. That is why CRISPR has become the most effective and highly applied system in various aspects by increasing food security through enhancing crops and nutritional value, resulting in a technology of choice for the world population [5].

Addressing these challenges, over the years, CRISPR has been increasingly incorporated into modern breeding methods, proving to be an effective tool for genome editing [6,7,8,9]. The delivery efficiency of CRISPR components into plant cells remains a technical challenge, limiting the full potential of CRISPR technology. Plant cells, unlike animal cells, possess a highly complex cell wall, which acts as a natural barrier that stops the CRISPR components from being easily introduced into the cell. Because of these natural physical barriers, delivery methods have developed and continue to develop so that the CRISPR components can be inserted into the cell more efficiently and the molecule can be transported into the nucleus safely to ensure precise editing of the genome [10].

Nanotechnology is emerging as a promising alternative method to improve the delivery of foreign molecules into plant cells. Nanoparticles have been studied more in animal cells than in plant cells in genome editing (GE) because animal cells do not possess a rigid cell wall. However, nanoparticles have gained ground in genome editing as an alternative method of integrating exogenous material into plant cells [11,12]. Because of obstacles presented by alternative delivery methods, genome editing has rapidly used advancing nanotechnology to transport exogenous genetic material into plant cells. NP could surpass the current challenges that other delivery methods have, enhancing the protection of the complex and the transformation efficiency [13].

### 1.1. Brief Overview of CRISPR Systems

CRISPR/Cas immunity encompasses three distinct phases: adaptation, expression, and interference. The CRISPR phases are interrelated with each other, and if any stage does not proceed as expected, it can lead to compromising the immune response to invasive nucleic acids, which can fail, increasing susceptibility to infections and, in some cases, even cell death [14,15,16].

Most of the six types derived from the two CRISPR classes are included in the adaptation phase. Adaptation, also referred to as insertion or the acquisition process, encompasses the selection, processing, and integration of short foreign DNA fragments into CRISPR arrays to generate new spacers from invading genomes. Most class 1 and class 2 systems contain the effector complexes along with the guide RNA (gRNA) and share the common genes, Cas1 and Cas2, which are involved in the adaptation process. In the adaptation process, two types of spacer acquisitions exist, named type I and II. Both types use the same complex to cleave and insert the spacer, which is (cas12-cas2)2. The fundamental difference is that in type I, DNA can bend due to the integration of the host factor, which binds the leader sequence. In type II, the leader anchoring sequence, which identifies the complex and the spacer, is inserted into the CRISPR array [17,18]. In the expression stage, the CRISPR array is transcribed into precursors of CRISPR RNA (pre-crRNA). During this stage, new spacers are gained from exogenous DNA, a process that is possible for most CRISPR types because protospacer adjacent motifs (PAMs) are near the protospacers. Unlike the other types, type III systems do not require recognition of PAM, but self-targeting is based on the complementarity between crRNA and the CRISPR array [18,19]. The interference stage is the step in which the foreign invasive nucleic acid sequence is cleaved by the Cas protein, which is activated and guided by crRNAs [20].

### 1.2. Current Transformation Methods

#### 1.2.1. Agrobacterium-Mediated Delivery Method

For the delivery of the CRISPR complex using Agrobacterium methods, two Agrobacterium species are used: *Agrobacterium tumefaciens* and *Agrobacterium rizogenes*. *Agrobacterium tumefaciens* is most commonly employed for delivering genetic material to obtain transgenic dicotyledonous and monocotyledonous plants. Agrobacterium-mediated transformation relies on the bacterium’s ability to transfer DNA from itself to the plant cell. The CRISPR cassette is introduced into the Agrobacterium’s T-DNA, which infects the plant through the wounds in the explant. The T-DNA containing the CRISPR cassette may become stably integrated into the host plant’s genome, potentially resulting in a genetically transformed plant; however, CRISPR-mediated genome editing can take place prior to stable integration of T-DNA in the plant genome. Agrobacterium can be used as a delivery method because of the virulence genes it possesses. These virulence genes are on a plasmid into which the CRISPR cassette is introduced, enabling the transfer of the DNA into the plant genome. This transformation method can be used for many plant species, but it has limitations; some plants are resistant to Agrobacterium or possess opposing resistance [21,22]. The Agrobacterium-mediated delivery method was first used in 1983 [23]. Since then, this method has been studied for decades, and today there are standardized protocols for this type of delivery [24].

#### 1.2.2. Particle-Bombardment-Mediated Delivery (Biolistic)

The term biolistic is derived from the abbreviation of biological ballistics and is also known as the particle bombardment or gene gun, a method that involves directly bombarding cells with DNA fragments using a gene gun. The particle bombardment delivery method (biolistics) is an alternative for genetic manipulation, employing a biological component as the carrier. This method was the first to achieve organelle transformation (in mitochondria and chloroplasts) and to transform recalcitrant crop species. The biolistic delivery method was developed to deliver DNA directly into cells. A major advantage of biolistics is its applicability to transferring DNA to both eukaryotic and prokaryotic organisms, including fungi, bacteria, algae, plants, and animal cells. The method works by accelerating a carrier particle (gold, wolfram, etc.) with a diameter of two microns, to which the engineered complex is bound. The carrier particle breaks the cell wall, and once the complex is inside, genetic manipulation begins [25,26,27,28,29].

#### 1.2.3. PEG-Mediated Delivery

Polyethene glycol (PEG)-mediated delivery is another of the most commonly used methods after Agrobacterium-mediated delivery and the biolistic method. Because of the advantages of the PEG method (low-cost, rapid process, no specialized equipment required, stable results, and no need for aseptic conditions), it has become a widely used technique for plant protoplast transformation [30]. PEG delivery involves the physicochemical uptake of foreign DNA by increasing plasma membrane permeability via endocytosis in plant cells or protoplasts, under the action of polyethene glycol, calcium chloride, and also high pH [12]. The polyethene glycol concentration is between 15 to 40%, along with other chemicals. After the process is complete, the PEG is removed, and the intact protoplasts are separated from the other chemicals and cultivated on regeneration medium [31,32]. The advantage over other methods is that the PEG-mediated delivery method can deliver ribonucleoproteins without traces of foreign DNA [29].

The main characteristics, advantages, and limitations of these delivery methods are summarized in Table 1.

#### 1.2.4. Limitations of Traditional Delivery

**Figure 1:**
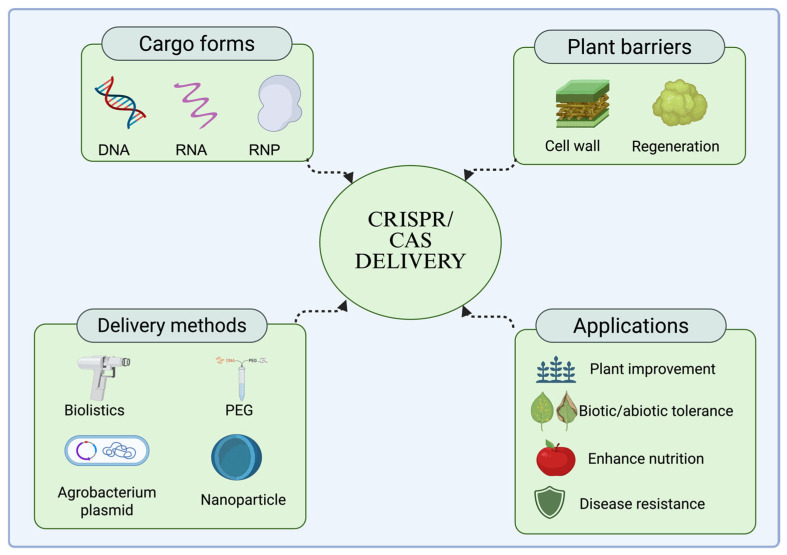
General components of CRISPR/Cas delivery in plants. Created in BioRender. Huiban, F. (2026) https://BioRender.com/pl7bf0a.

Plants have complex genomes and structures compared with other living organisms. Genome editing has been conducted in a limited number of plant species due to restrictions in the current traditional methods restricted. The most widespread methods to deliver the CRISPR components into plants are Agrobacterium-mediated delivery and particle-bombardment-mediated delivery (biolistics), where the CRISPR cassette is integrated randomly into the cell, with possible continuous expression in the host cell. The Agrobacterium method is one of the principal methods used in plant genome editing; it has limitations in its applicability to a broad range of species (Table 1). The limitation of the host range reduces its applicability, with several species being recalcitrant to this type of delivery method. The floral dip method also has restricted applicability; it is accessible to *Arabidopsis thaliana* and related species [21]. The major limitation of the particle-bombardment-mediated method is that delivery of the CRISPR components is inaccurate, leading to potential genome-wide damage through brute force and the use of chromosomal rearrangements [24]. An overview of the major CRISPR/Cas delivery strategies in plants is presented in Figure 1.

**Table 1:** Comparative analysis of delivery methods for plant genome editing.

Delivery Method	Transformation Efficiency	Host Range	Transgene Integration	Tissue Damage	Regeneration Requirement	Scalability
Agrobacterium-mediated transformation	High [21,22,23,24]	Moderate-high [21,22]	Frequent [21,22]	Moderate [33]	Required [34]	High [22,32]
Particle bombardment (biolistics)	Moderate-high [25,26,27,28,29]	Broad [25,26,27,28,29]	Frequent [25,26,27,28,29]	High [24]	Required [34]	Moderate [31]
PEG-mediated delivery	Moderate [30,31,32]	Limited [30,31,32]	Low [29,30,31]	Low [29]	Required [31]	Moderate [35]
Nanotechnology mediated delivery *	Moderate [36,37]	Broad (potential)	Moderate (potential) [10]	Low [38]	Reduced [4]	High [2]

Note: * Currently under active development and the performance may vary.

## 2. CRISPR/Cas Systems Used in Plants

### 2.1. CRISPR/Cas9

CRISPR/Cas9 has become the most used system in plant genome editing. Type II Cas9 from *Streptococcus pyogenes* has two elements: the Cas9 nuclease combines with a single guide RNA (sgRNA), which is an engineered fusion of crRNA and a fixed transactivation crRNA. Because of this, the CRISPR/Cas9 complex can be used as an effective tool to target specific DNA sites. In the context of delivery, CRISPR/Cas9 can be introduced into the cell as plasmid DNA, RNA, or a ribonucleoprotein complex (RNP), providing versatility for nanoparticle-based delivery [39]. Cas9 has been proven to be effective in several species. In rice (*Oryza sativa*), three SWEET genes have been targeted for the outcome of bacterial blight resistance by using the CRISPR/Cas9 system [40]. Cas9 was used in wheat (*Triticum aestivum*) for powdery mildew resistance [41], in maize (*Zea mays*) to improve yield stability [42], and soybean (*Glycine max*) to improve the quality of oleic acid content [43]. In forestry, CRISPR/Cas9 was used on *Populus trichocarpa* and *Eucalyptus grandis* to change the lignin biosynthesis pathways [44,45] and in various other plant species.

### 2.2. CRISPR/Cas12

Cas12, also known as Cpf1, is classified as a class 2 type V system. Cas12 functions similarly to Cas9 but recognizes different PAM sequences, and it uses a single CRISPR RNA (crRNA) to recognize and cleave the targeted DNA sequence [46]. Cas12, like Cas9, can be delivered in a multitude of molecular arrangements; its functioning relies on crRNA instead of sgRNA, presenting a reliable option for nanoparticle-based delivery into plants [47,48]. Cas12 has proven its efficiency in several major plants, including rice (*Oryza sativa*), tobacco (*Nicotiana tabacum*), for efficient targeted mutagenesis [46], tomatoes (*Solanum lycopersicum*), Arabidopsis (*Arabidopsis thaliana*), and wild tobacco (*Nicotiana benthamiana*) for targeted mutagenesis [49]. In 2020, in forestry, CRISPR/Cas12 was first used on the *Populus alba* × *Populus glandulosa* hybrid, where three Cas12 nucleases were tested, with AsCas12a showing the highest mutation efficiency at 70%. Before this, the effectiveness of Cas12 nucleases in woody plants was unknown [50].

### 2.3. Base Editors

Base editors are represented by advanced engineered CRISPR systems that substitute nucleotides without using foreign donor DNA [51], facilitating direct substitutions for targeted nucleotides from cytosine (C) to thymine (T) and from adenine (A) to guanine (G) for endogenous genes [52]. The base editors are divided into two distinct classes: cytosine base editors (CBEs) and adenine base editors (ABEs). Cytosine base editors (CBEs) are used to make substitutions from C-G to T-A, while adenine base editors can convert G-C to A-T. The base editors are composed of CRISPR/Cas9 variants, sgRNA, and deaminases, which increase the size of the molecule, making the cargo complexity for nanoparticles higher than that of the Cas9 protein [53]. Base editing is applied in many species to remodel different endogenous genes. For instance, in rice (*Oryza sativa*), different CRISPR/Cas9 variants were applied to create substitutions for cytosine and adenine simultaneously in OsSPL sites. Similar approaches were done in tomato (*Solanum lycopersicum*) and potato (*Solanum tuberosum*) [54], Arabidopsis (*Arabidopsis thaliana*), rapeseed (*Brassica napus*) [55], and a hybrid poplar clone (*P. tremula* × *P. alba* clone INRA 717-1B4) [56,57], and Citrus (*Citrus sinesis*) [57].

### 2.4. Prime Editor

Prime editors are a versatile genome editing technique that use reverse transcriptase, which may reduce undesired off-target editing in plant genomes and enhance the efficiency of specific insertions by utilizing double-stranded break (DSB)-free procedures [58,59]. Delivery of prime editors via nanoparticles is exceptionally difficult because of the molecule’s multifaceted structure, which includes a Cas9 nickase, reverse transcriptase, and pegRNA [59]. Prime editing has been effectively implemented in several plant species, including rice (*Oryza sativa*) [60], maize (*Zea mays*) [61], wheat (*Triticum aestivum*) [62], peanut (*Arachis hypogaea*), chickpea (*Cicer arietinum*), cowpea (*Vigna unguiculata*) [63], tomato (*Solanum lycopersicum*) [64], and other crop species. In forestry, currently, a poplar hybrid (*P. alba* × *P. glandulosa*) is the only documented species where prime editing was demonstrated [65]. Despite the evidence that prime editing is an advanced tool used in genome editing, its efficiency is rather low, which is influenced by parameters such as pegRNA, playing a key role in the efficiency of prime editing, the reverse transcriptase template, the prime binding site, the repairing pathway, and the location of the selected region to induce mutations [66].

## 3. Challenges in CRISPR Delivery in Plants

### 3.1. Cell Wall Barrier

The plant cell wall is a multilayer rigid physicochemical barrier that protects the cell from external influences. In genome editing, the plant cell wall is a major bottleneck that lowers the possibility of delivering DNA, RNA, or RNP, which slows down progress in genetic engineering. Due to this, specific delivery methods have been developed to overcome this challenge. The two most common delivery methods used in plants are Agrobacterium-mediated delivery and biolistics (gene gun), both of which can surpass the natural barrier of the cell due to their unique characteristics, but these methods also have disadvantages. Currently, genome editing in plants lacks an exogenous genetic material delivery method that allows a passive delivery with minimal or without causing damage to the cell [10,67]. In crops, the genetic transformation progress is more prominent than in other species [68], such as woody plants, which remain more recalcitrant due to genotype dependency and regeneration difficulties [69], and highly developed cell walls (lignin, cellulose, and hemicellulose) with increased lignification [70].

### 3.2. Low Transformation Efficiency

Multiple biological factors influence the transformation efficiency, and delivery methods remain closely connected to the effectiveness of CRISPR components within cells, especially for large molecules such as RNP. Effective transformation efficiency has been achieved in several species with different delivery methods; each delivery method has different transformation efficiencies [10]. Agrobacterium-mediated delivery offers stable transformation efficiencies, but this method is efficient only in a narrow variety of species. However, the regeneration process of transformed cells is sophisticated, often resulting in no results. The particle bombardment method offers broader applicability than the Agrobacterium delivery method. Many factors play a key role in the efficiency of this delivery method, such as the particle type used, quantity, size, and acceleration process, and the transformed cells are usually damaged because of the force applied, which leads to a high probability of the regeneration of tissue [71]. In crop species, the transformation efficiency can vary from below 1% reported in soybean (*Glycine max*), the lowest transformation efficiency [72], to the highest of 75% in *Nicotiana tabacum* [73]. In forestry, the efficiency differs from the crop species; the lowest value reported is 1.9% in *E. urophylla* × *E. grandis* [74], and the maximum is above 50% in *Populus alba* × *P. glandulosa* [75].

### 3.3. Tissue Culture Dependency

Tissue culture dependency is recognized as a bottleneck that limits transformation efficiency; most of the delivery methods used in plants require the transformed cells to be regenerated into whole plants. A successful CRISPR transformation does not only require an effective delivery of the CRISPR cargo into the cell, but also an optimized tissue culture, depending on the delivery method used and also on the type of explant and species [76]. For regulating organogenesis in vitro, two widely used phytohormones are used; different concentrations of auxins and cytokinins have been studied over the years on several species to induce callus formation leading to whole-plant regeneration, which is a critical step to a successful transformation [77]. Many types of tissue cultures can be achieved; a few examples are immature embryos, axillary buds, young leaf segments, pollen, internodal segments, root segments, and others. For each type of explant used, specific protocols must be developed to achieve successful transformation [78].

### 3.4. Off-Target Effects

One challenge genetic manipulation often faces is off-target effects, which affect the development of CRISPR in plants. In many plants, compared with other cell types, the frequency of off-target effects is lower, making the genome-editing tool CRISPR more effective in plants than in human cells [79]. Off-targets are a common challenge in genome editing. Inducing off-targets in a genome can lead to chromosomal rearrangements, causing mismatches and completely changing the genome. If the off-target is not kept under control, it can lead to unintentional changes in different genes, which can cause the genes to lose their functions or change them, ensuring that the plant develops physiologically differently from the parent plant [80]. Off-target effects have been classified into three types. The first type refers to regions and other PAMs (5′-NGG-3′) containing substitutions or mismatches. The second type refers to indels in the regions and other PAMs (5′-NGG-3′). The third type involves the removal of sequences with various PAMs (5′-NAG-3′) [81]. The design process is one of the most important steps in reducing the number of undesired off-target effects. Cas9 and Cas12a are highly specific, but undesired off-target effects can still appear. By designing highly specific single-guide RNA (sgRNA), the number of undesired incidents within the genome can be drastically reduced [82]. Off-targets mutation has been reported in many species; a few examples in crops are *Oryza sativa* [83], *Vitis vinifera* [84], *Arabidopsis thaliana* [85], etc., but less in woody plants because tree species were not all sequenced and studied at the time; off-targets were reported in *Populus tremula* × *alba* and *Eucalyptus grandis* × *urophylla* hybrids [86].

### 3.5. Delivery of CRISPR Components

The delivery of the CRISPR complex can be made with several methods; the methods are chosen depending on the type of cells that are used and the desired result. The challenges for the delivery of the CRISPR complex are the optimization and engineering of the chosen method for the desired species. Also, there is a need to develop standards for all the methods in both in vivo and in vitro assays, as well as less costly and more efficient methods to engineer CRISPR delivery vehicles. Most of the methods depend on specialized equipment, limiting the scalability and ease of the implementation of the methods. All these challenges affect CRISPR and genome editing, restraining the method from being applied in most cases, only in research, and less in large-scale industries [87].

## 4. Nanotechnology-Based CRISPR Delivery Systems

Nanotechnology has developed in the late twentieth–early twenty-first century [88] as a need to address several limitations in plant biotechnology. Nanotechnology has entered the field of agroforestry to deliver pesticides (nanopesticides), fertilizers (nanofertilizers), and overall to deliver different chemicals (nanochemicals) and nanobiosensors [89,90]. Recently, nanotechnology has attracted attention in the scientific world in the field of genome editing, due to its ability to deliver different molecules effectively, with the potential to overcome the classic bottlenecks found in traditional gene delivery methods, such as the low transformation efficiency in specific species and the thicker cell wall found in woody plants, where the current delivery methods have difficulties penetrating the cell wall and are subject to genotype dependency [91].

**Figure 2:**
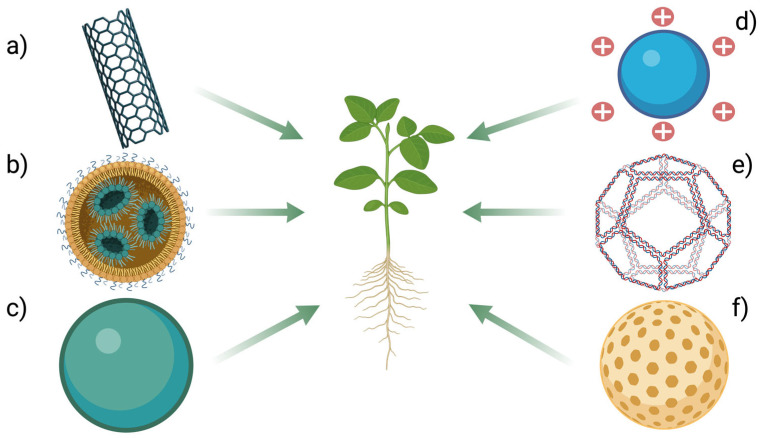
Types of nanoparticles used in plant biotechnology. (**a**) carbon nanotube, (**b**) mesoporous silica nanoparticle, (**c**) green nanoparticle, (**d**) magnetic nanoparticles, (**e**) DNA nanostructures, (**f**) lipid nanoparticles. Created in BioRender. Huiban, F. (2026) https://BioRender.com/0fjswlo.

Nanotechnology, through its capacity to modify matter at the nanoscale, offers high efficiency, durability, and versatile surface chemistry, and has the potential to transform many areas of plant biotechnology [36]. Nanoparticles (NPs) or nanomaterials (NMs) are very small particles ranging from 1 to 100 nm [92] and hold the ability to enter plant cells, passing through the cell wall pores without the use of brute force [24]. Nanotechnology-based CRISPR includes carbon nanotubes (CNTs), mesoporous silicon nanoparticles (MSNs), lipid nanoparticles (LNPs), magnetic nanoparticles (MNPs), and green nanoparticles, and has been used to deliver different components into the cell, such as RNPs, DNA, RNA, and proteins [21]. A visual representation showcasing the nanoparticles is presented in Figure 2.

### 4.1. Carbon Nanotubes

Carbon nanotubes (CNTs), as the name suggests, are carbon-based nanomaterials that are shaped in the form of tubes. CNTs are divided into two classes based on the number of concentrical walls: single-wall carbon nanotubes (SWCNTs) and multi-walled carbon nanotubes (MWCNTs) [4]. SWCNTs have a diameter of 0.6 to 2.0 nm [93] and comprise a singular layer of graphite in a cylindrical configuration, whereas MWCNTs with a diameter of 5 to 100 nm [94] are composed of various layers of concentric graphite [95]. Carbon nanotubes have been shown to have the ability to deliver DNA and RNA into cells. Besides being able to deliver genetic information, it is a viable option to deliver plasmid DNA and the CRISPR/Cas9 complex, conferring protection from enzymatic degradation for the molecules it carries [96]. Combining CNTs with different molecules, particularly polymers (polyethylenimine), arginine, or chitosan, enhances the nanotubes’ ability to load DNA more efficiently. In a recent study, the carbon nanotube-mediated delivery of plasmid DNA into cowpea leaves was achieved, causing no toxicity or damage to the tested leaves [38].

### 4.2. Lipid Nanoparticles

Lipid nanoparticles (LNPs) are a delivery method composed of four kinds of lipids: ionizable lipid, PEG-lipid helper phospholipid, and cholesterol, forming the lipid nanoparticle structure [97]. LNPs, because of their structure, are composed of a hydrophilic head and a hydrophobic tail; this structure offers an ideal intercellular delivery of CRISPR/Cas9 into the cell [98]. LNPs have a diameter of <100 nm and consist of lipids and the nucleic acid cargo [99]. A ribonucleoprotein (RNP) with an overall negative net charge is produced by complexing the cationic Cas9 protein with a net charge of +22 with gRNA. For the encapsulation of all CRISPR/Cas9 components (plasmid DNA, mRNA, or RNP), ionizable cationic lipids are consequently essential [100].

### 4.3. DNA Nanostructures

DNA nanostructures are artificially designed structures engineered by modifying the sequence, length, and topology of DNA strands, allowing the development of different DNA structures such as cubes, tetrahedra, and polyhedral structures. The advancements in computational design tools have revolutionized the precise regulation of DNA nanostructure assembly, facilitating the creation of complex designs, such as DNA origami, DNA tiles, and dynamic nanomechanical devices [101]. DNA possesses the unique ability that the nucleotides can be precisely designed, making it possible to rearrange the DNA into unique complex two-dimensional (2D) and three-dimensional (3D) structures [102]. Two-dimensional nanostructures can be formed via rolling circle amplification (RCA), hybridization chain reaction (HCR), exponential isothermal amplified strand displacement reaction, and catalytic hairpin assembly [103]. Sun et al. developed a DNA nanoclew vehicle to deliver CRISPR components in mice; the vehicle was a yarn-like structure synthesized by rolling-circle amplification (RCA) [104]. DNA nanostructures have been studied less in plants for genome editing than in other fields. Zhang et al. have successfully shown that DNA nanostructures can be used as an effective delivery vehicle for siRNA in plant cells for efficient gene silencing [105].

### 4.4. Mesoporous Silica Nanoparticles 

Mesoporous silica nanoparticles (MSNs) are made of silicon dioxide (SiO_2_) [96], characterized by pores with diameters between 2 and 50 nm [93]. Different methods can be used to synthesize MSNs, such as sol-gel synthesis, hydrothermal synthesis, microwave synthesis, template synthesis, and soft and hard templating. Each method used has a different result on the morphology of MSNs, resulting in nanospheres, yolk-shells, hollow porous structures, and core-shells, regulating the surface area, encapsulation efficiency, and biomolecular release behavior. Cationic MSNs can bind electrostatically with DNA or RNA, forming a complex that protects the molecules, enhancing the stability and cellular uptake [106]. MSNs can successfully penetrate the cell wall, resulting in a high efficiency of gene delivery [37] and can incorporate different nucleic acid cargos of different sizes, from siRNA to larger molecules such as CRISPR/Cas complexes [107]. Xu et al. demonstrated that MSNs can deliver a CRISPR/Cas9 plasmid into U2OS cells, reaching a loading efficiency of approximately 50% [108].

### 4.5. Magnetic Nanoparticles

Magnetic Nanoparticles (MNPs) are usually nanoparticles coated with iron oxide and cationic polymer polyethyleneimine (PEI), which is a method to deliver exogenous genetic material into the targeted cells using magnetic fields by aligning the vector with the MNPs through a process called magnetofection [94,109,110]. Mainly, magnetofection has been applied in medical and animal research, slowly emerging into plant sciences. Plant cells have a naturally developed cell wall, acting as a natural barrier against incorporating foreign DNA, making them less susceptible to genome editing, in contrast with bacterial and mammalian cells, which lack this type of defensive mechanism [111]. Recently, magnetofection was reported in both dicotyledons (okra [112], tabaco [100], cucumber [113], pumpkin, and pepper [111]) and monocotyledons (maize, sorghum [114], and lily [115]). However, the pollen of some species is less appropriate to magnetic nanoparticle transformation along with targeting specific organelles such as chloroplasts and mitochondria [116].

Pollen magnetofection uses cationic polyethyleneimine (PEI) coated with Fe_3_O_4_ magnetic nanoparticles serving as a carrier for the delivery of DNA. The positively charged nanoparticles bind with the negatively charged DNA molecule, resulting in the formation of a stable MNP-DNA complex. Applying external magnetic fields to the MNP-DNA complex towards the pollen enhances penetration through the pollen before pollination [111].

Magnetic nanoparticles have been used to genetically modify pollen and have enabled a tissue culture-free procedure when magnetofection is combined with pollen-mediated transformation. Because this technique is mainly performed on pollen and limited processes on plant tissues, it could be applied to a wide range of species and not be species-dependent like other delivery methods. Even though it has been proven to deliver the CRISPR/Cas9 plasmid and could potentially transform a variety of species, the transformation efficiency varies between species [4,117].

### 4.6. Green Nanoparticles

Green nanoparticles can be synthesized from various sources, such as bacteria, yeast, or plants; the most commonly used source is plants because they are easily and widely available, offering also a much faster and more stable method than other methods [118]. Chitosan-based nanoparticles are one of the green methods used that can deliver exogenous DNA/RNA, as well as the CRISPR/Cas9 components. Chitosan has a series of attributes that have garnered attention, such as biodegradability in the cell, biocompatibility, and other biological activities. Based on the purpose, chitosan-based non-viral vectors can be synthesized into various forms and shapes, ranging from nanoparticles to micelles, each with distinct characteristics [87,119,120]. The application of green nanoparticles remains limited as a delivery method in plants; research in the field on green nanoparticles as a delivery method for CRISPR/Cas9 in plants remains largely unexplored (Figure 2).

Even though there are several types of nanoparticles, they are included under the umbrella of the same technology; they are quite different from each other in terms of size, operating strategy, loading cargo capacity, release mechanisms, cellular uptake pathways, and toxic effects. The size of nanoparticles is usually less than 100 nm. In the case of SWCNTs, the diameter is <2 nm, enabling them to fit very small molecules. Due to the small diameter of the nanoparticles, they can be delivered in a variety of approaches, such as leaf infiltration, the spraying method, the magnetic field-assisted method, and the ultrasound-assisted method, and other approaches [94]. From a toxicity point of view, carbon nanotubes (CNTs) have the potential to induce phytotoxicity effects by inducing oxidative stress, affecting the biological processes of plants [121]. Also, the physicochemical properties of NPs affect the delivery in plants. The surface charge, along with the size, allows NPs to pass through the cell wall through the pores [94].

## 5. Advantages of Nanomaterial-Based Delivery

Traditional delivery methods, Agrobacterium-mediated transformation, and the biolistic particle delivery method, despite their limitations, are among the most widely used in plant genomics. The main challenges that the traditional methods have are random DNA integration into the cell, causing physical damage, and being species-dependent. The novel nanotechnology-based delivery method reduces cell damage and offers high versatility because these nanomaterials can be engineered to deliver precisely the desired molecules into plant cells [12]. Although nanomaterial-based delivery in plants has not been as extensively studied as in animal and medical research, studies in the field provide essential knowledge for applying this technology to plant genome editing, particularly when combined with the CRISPR system.

Nanotechnology-based delivery offers many particles that can be designed to serve as the delivery vehicle for the complex into the cell. Nanoparticles offer high surface-to-volume ratios and surface functionalization possibilities. These features allow for cargo preservation and controlled biomolecule delivery, making them viable alternatives to standard transformation techniques. These techniques protect plants from various stresses, becoming an alternative to traditional delivery methods [122].

MSNs enable high adaptability for a large amount of DNA and proteins, such as CRISPR/Cas9, releasing the cargo at the target site in a controlled manner. MNPs also have several advantages over the conventional methods; they can protect the cargo from digestion, offering better penetration of the cell wall due to transporting the biomolecules under the influence of an external magnetic field, enhancing the genetic material load capacity, and providing a relatively low-cost delivery system [123].

A nanotechnology-based delivery method is evaluated using multiple performance metrics, including delivery, transformation, and genome editing outcomes. Transformation efficiency, defined as stable genomic integration, can be high in some cases; it can approach 100% in stable transgenic plants. However, off-target mutations associated with CRISPR/Cas9 pose a significant limitation [116]. Gene editing efficiency represents targeted genome modification after delivery of the genetic material/CRISPR components into the cell. Gold nanoparticle-based delivery achieved approximately 80% delivery efficiency of the NPR1 gene in *Arabidopsis thaliana* [124]. Nanotechnology-based delivery systems in plants exhibit variable but often substantial cargo delivery efficiency. Carbon nanotube (CNT)-mediated delivery has demonstrated internalization efficiencies of approximately 62% in plant leaf cells [10]. Beyond cellular uptake, NPs can exhibit different cargo delivery efficiencies and have been proven to be effective in guard cells, the extracellular space, and in chloroplasts [125]. Overall, the nanotechnology-based delivery method is a promising delivery approach, offering a variety of transformations, deliveries, and editing outcomes. It is necessary to mention that these values vary from species to species and under experimental conditions, and the protocols for nanoparticle-based delivery have not yet been standardized within plant biotechnology. It is necessary to deepen research on this method in plants; its large-scale application in plant biotechnology remains insufficiently explored, unlike in other fields of research.

Notwithstanding these advantages and applications of nanoparticle technology, nanoparticle-based methods of delivery face limitations regarding tissue toxicity, transformation efficiency, and environmental impact, emphasizing the necessity for more research resulting in the optimization of the process prior to their extensive use in plants [122].

## 6. Applications in Agroforestry Improvement

The CRISPR/Cas system has been widely used in many plant species to enhance specific traits, including resistance to biotic and abiotic stresses, yield, and nutritional value. Expression analysis is the first step to identify and select the genes for GE when designing the gRNA in CRISPR/Cas technology [126]. In *Oryza sativa*, resistance to bacterial blight was achieved by using CRISPR to edit the OsSWEET genes [40]. Additionally, Bsr-d1, Pi21, and ERF922 were genetically modified to promote resistance to rice blast caused by *Magnaporthe grisea* [127]. In *Triticum aestivum*, CRISPR was used to develop wheat resistant to wheat dwarf virus [128]. CRISPR/Cas9-mediated mutagenesis was applied in tomato (*Solanum lycopersicum*) to target the RIN gene, improving fruit ripening and shelf life [129]. CRISPR was also used in potato (*Solanum tuberosum*) to reduce enzymatic browning by editing the Polyphenol Oxidase gene (PPO) [130], and in soybean (*Glycine max*) to alter fatty acid composition by targeting Fatty Acid Desaturase 2 (GMFAD2-1A and GmFAD2-1B), resulting in higher oil quality [43]. These studies highlight the versatility of the CRISPR/Cas system in developing a variety of traits across species.

Notwithstanding these advantages and applications of CRISPR in plants, the delivery of CRISPR components remains a key limitation, especially for recalcitrant species. Nanotechnology-mediated delivery is increasingly studied to overcome the barriers posed by other delivery methods. Carbon nanotubes mediated delivery to effectively deliver plasmid DNA into intact tissues of *Nicotiana benthamiana*, *Triticum aestivum*, *Eruca sativa*, and *Gossypium hirsutum* without using external force with an efficiency of as high as 86% [10,91]. Mesoporous silica nanoparticles have been employed as an effective carrier for nucleic acid delivery in plants, supporting transgene expression in *Nicotiana tabacum*, *Zea mays* [131], and in *Arabidopsis thaliana* [132]. Magnetic nanoparticles, also known as pollen magnetofection, offer a non-invasive delivery method of gene transfer and have been applied in *Cucumis sativus* [113], *Lillium regale* [115], *Capsicum annuum* L., *Cucurbita pepo* L., *Lilium brownii*, and *Cucurbita moschata* [111]. Although applications of nanoparticle-mediated delivery of different cargos into plants have been demonstrated, in forest biotechnology, their applications remain largely unexplored, highlighting the research gap in genome editing of tree species.

Nanomaterial-based methods hold tremendous potential to overcome the bottlenecks inherent to traditional delivery methods, and their application is gradually expanding to genome-editing technologies in plants. It is important to note that CRISPR, when combined with nanoparticle-mediated delivery, has not been widely established and its applications remain limited in plants. Their application is presented in Table 2.

**Figure 3:**
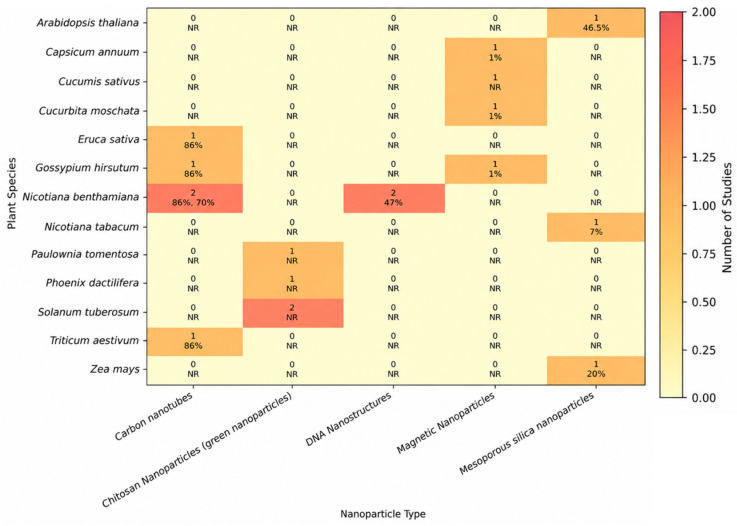
Current status of nanoparticle-mediated delivery systems in plants: distribution of studies and reported efficiencies across species for nanoparticle platforms. Upper value = nr of studies; Lower value = transformation efficiency (%); NR = not reported.

Figure 3 highlights the current distribution of nanoparticle-mediated delivery studies across plant species and nanoparticle classes based on Table 2. The two types of delivery systems with the greatest extent of investigation and number of applications in the literature are: chitosan nanoparticles and carbon nanotubes. Regarding the number of delivery systems that have been studied, *N. benthamiana* is clearly the most widely used experimental system across studies involving both CNTs and DNA nanostructures for use as a model species for studies related to molecular delivery. CNTs are reported to have some of the highest delivery efficiencies, with at least one example of efficiency as high as 86%, while for DNA nanostructures, at least one study reported an efficiency of 47% for gene silencing in *N. benthamiana*. Chitosan NPs (green NPs) have been used primarily in crop and woody species (i.e., including *Solanum tuberosum*, *Paulownia tomentosa*, and *Phoenix dactylifera*), providing evidence to suggest their suitability for transformation applications in plant species outside of model plants. In contrast, MNPs and MSPs have been evaluated in a very limited number of species, and therefore, delivery efficiencies for magnetic nanoparticles and mesoporous silica nanoparticles are typically reported between approximately 1 and 20%. The heat map shows that the majority of studies have been conducted based on a few models and crop species; however, few of these types of studies have been conducted in forestry species, showing a considerable gap in the research area, which represents a substantial opportunity for the development of nanoparticle-mediated delivery systems in forest biotechnology.

**Figure 4:**
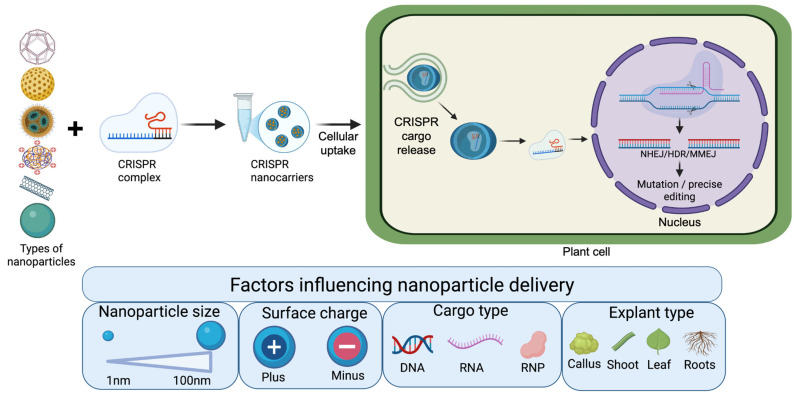
Nanoparticle-mediated CRISPR/Cas delivery in plants: workflow and factors influencing editing efficiency. Created in BioRender. Huiban, F. (2026) https://BioRender.com/z6h4af3.

Figure 4 provides a representation of the operational flow and the contributing factors to different nanoparticles that can be used for nanoparticle-mediated CRISPR delivery. The CRISPR complex, after being encapsulated within CRISPR-nanocarriers and introduced into the cell, benefits from nanoparticle protection. This protection is maintained until the nanocarrier is released prior to nuclear entry, facilitating genome editing and subsequent DNA repair via the intended mechanism. Beyond the nanoparticle type, a range of additional elements affects delivery efficiency. The passage of CRISPR nanocarriers through the cell wall is affected by nanoparticle size, while cell membrane interaction is mediated by surface charge and nanocarrier stability. Cargo types also influence delivery; different cargos have specific sizes and require potentially specific nanocarrier protection. The delivery method elicits differential responses from explant types such as callus, shoots, leaves, and roots, each demanding particular environmental conditions.

## 7. Biosafety and Regulatory Considerations

For CRISPR-edited plants, biosafety and regulatory considerations remain complex and rapidly changing, especially when CRISPR editing is combined with a nanoparticle-based delivery system. The European Union has recently made new proposals on regulating new genomic techniques (NGTs), aiming to differentiate genetically modified organisms (GMOs) by dividing them into two categories based on the type of genetic modification [139]. The United States has supported the application of CRISPR technologies to plants and has chosen a product-oriented approach where GMO regulation does not apply to crops with minimal modification, leading to a series of financial advantages [140]. Each nation has a different approach when it comes to biosafety and regulation considerations of GMOs, advancing towards developing guidelines about implementing regulations based on the type of genetic modification [141]. Japan, India, Australia, and countries from North and South America have specific regulations for genetically edited crops, with edited crops being sold on the market. In the case of the European Union, the regulations are more severe for genome-edited plants; the cultivation of genome-edited crops is a difficult process that needs authorization [142]. The first CRISPR-edited food was sold directly to consumers in Japan in 2021. The sold product was Sicilian Rouge tomatoes, which are genetically enriched to have a high amount of γ-aminobutyric acid (GABA). The tomatoes were first tested on 4200 farmers who requested the seedlings, and after that, because of the high demand, the fresh tomatoes were sold directly to customers [143]. The regulations, considerations, and biosafety in forestry are more complex due to the long-life spans of the species, causing long-term monitoring of the environment [144].

While countries have developed regulations to address genome editing, nanoparticle technology remains slightly uncovered. Regulatory bodies worldwide are currently addressing these issues by developing guidelines for nanotechnology [145]. Nanotechnology research has been shown to impact the environment and different industries, which emphasizes the necessity of biosafety standards for nanotechnology, especially when it is integrated with CRISPR.

## 8. Future Perspectives

### 8.1. Smart Nanoparticles

It is anticipated that nanoparticle-based delivery would significantly enhance the delivery of the CRISPR system into plants; this type of delivery was also developed as a smart nano-delivery system [146]. Nanoparticles can be engineered as a delivery cargo for siRNA into mature plant tissues to induce gene silencing without the demand of transgenic integration [134]. Several nanoparticles, such as carbon nanotubes (CNTs), mesoporous silica nanoparticles (MSNs), magnetic nanoparticles (MNPs), and other types of nanoparticles, have been demonstrated to have broad applicability in delivering exogenous genetic material in plants, which is a distinct advantage compared with the traditional delivery methods used in genome editing [97]. Although smart nanoparticle delivery offers unique advantages in the biomedical applications of CRISPR/Cas9 delivery, where smart nanoparticles respond to endogenous and exogenous signals, the same systems remain uncovered in plant biotechnology [147].

### 8.2. AI-Guided Delivery Systems

Computational algorithms that learn from data to recognize patterns and make predictions based on the data provided are a component of Artificial Intelligence (AI), including machine learning (ML) and deep learning (DL) [148]. The rapid development of Artificial Intelligence (AI) has infiltrated many areas; slowly, AI is paving its way into plant biotechnology, especially through the design, prediction, and interaction of nanoparticles. AI can help optimize the best design for the components that are transferred into the cell by successfully helping researchers develop new Ca9 variants, different from the wild type, while still maintaining their normal functions [149]. AI-driven models can predict how nanoparticles affect functional responses in plant cells, test different experimental conditions, and simulate interactions [150].

## 9. Conclusions

Various delivery methods are being developed to introduce cargo, along with exogenous DNA, into cells. The traditional delivery methods have proven successful in penetrating the plant cell wall and introducing the CRISPR/Cas9 complex into several plant species. However, CRISPR is a powerful advanced technology designed to genetically modify genomes, but its efficiency is limited because of the delivery methods, which, besides others, become a major overwhelming bottleneck. The Agrobacterium-mediated delivery method remains the most used method to deliver the complex, but one of the major challenges for this method is that certain species are recalcitrant to Agrobacterium, and tissue culture dependency. The second most used method is biolistics, which can penetrate the cell wall with the force of gold or tungsten microparticles at high velocity using a “gene gun”; the major challenge with this method is to deliver the complex without causing major damage to the cell, as transformed plant cells cannot regenerate a whole plant.

Now, emerging alternative methods to traditional delivery systems represent a promising future direction in GE, as they enable nanoparticle-mediated delivery. Notwithstanding, nanoparticle-mediated CRISPR delivery is still in its early stages. Its applicability has been more focused on mammalian cells and less on plants due to key challenges related to cargo loading capacity, cell wall penetration, delivery specificity, and environmental safety. The nanoparticle method has been proven to be able to deliver DNA, RNA, or RNPs. Nanoparticles have the ability to protect the cargo from digestion and have the potential to deliver bigger proteins, such as CRISPR/Cas9. Nanoparticles with the help of Artificial Intelligence can be engineered to be biodegradable and plant-compatible, which is a key condition in the biosafety of genome editing applications.

The future of this technology relies on the combination of different nanotechnology platforms with genome-editing tools. An engineered multifunctional nanocarrier can deliver exogenous genetic material through a controlled process to a specific part of plant cells, resulting in an improved delivery process of CRISPR/Cas technology. Nanoparticle-assisted delivery has been proven to be effective in a few crop species and is largely understudied in tree species. Nanotechnology and genome editing continue to develop together, and as they do, the fusions of these two areas may create new generations of precision breeding tools that will aid in the development of sustainable agricultural methods, resilient forestry practices, and future global food and environmental security.

## Figures and Tables

**Table 2:** Reported applications of nanoparticle-based delivery platforms in plant biotechnology.

NP Types	Species	Application	Attempts	Outcome	Transformation Rate	Reference
Carbon nanotubes	*Nicotiana benthamiana*, *Eruca sativa*, *Triticum aestivum*, *Gossypium hirsutum*	Genetic material delivery	2	Efficient DNA delivery without transgene integration	86%	[10]
	*Nicotiana benthamiana*	Gene slicing		GFP knockdown	70%	[133]
DNA Nanostructures	*Nicotiana benthamiana*	Gene silencing	2	Delivery of siRNA into mature plant tissues	47%	[105]
	*Nicotiana benthamiana*	siRNA delivery		Gene silencing	-	[134]
Mesoporous silica nanoparticles	*Arabidopsis thaliana*	Gene expression	3	Transient gene expression of intact roots	46.5%	[132]
	*Zea mays*	Protein delivery		Removing loxP-defined DNA fragment from genome	20%	[135]
	*Nicotiana tabacum*	DNA delivery		DNA delivery in plant cells and leaves	7%	[131]
Magnetic Nanoparticles	*Gossypium hirsutum*, *Capsicum annuum*, *Cucurbita moschata*	Pollen magnetofection	2	Transgenic plants through pollen magnetofection	1%	[111]
	*Cucumis sativus*	Pollen magnetofection		Producing transgenic seeds	-	[113]
Chitosan Nanoparticles (green nanoparticles)	*Solanum tuberosum*, *Paulownia tomentosa*	Gene transformation	3	Transformation of UidA and GUS gen	-	[136]
	*Solanum tuberosum*	Thionin gene transfer		Enhance resistance against fungus	-	[137]
	*Phoenix dactilifera*	Thionin gene transfer		Enhance resistance against fungus	-	[138]

## Data Availability

No new data were created or analyzed in this study. Data sharing is not applicable to this article.

## Abbreviations

The following abbreviations are used in this manuscript:

AI	Artificial intelligence
ABEs	Adenine base editors
CNTs	Carbon Nanotubes
CBEs	Cytosine base editors
CRISPR	Clustered regularly interspaced short palindromic repeats
DL	Deep learning
DNA	Desoxyribonucleic acid
DSBs	Double stranded breaks
GE	Genome editing
GMOs	Genetically modified organisms
LNP	Lipid nanoparticle
ML	Machine learning
MNPs	Magnetic nanoparticles
MSNs	Mesoporous silica nanoparticles
NGTs	New genomic techniques
NMs	Nanomaterials
NPs	Nanoparticles
PAM	Protospacer adjacent motif
PEG	Polyethene glycol
RNA	Ribonucleic acid
RNP	Ribonucleoprotein

## References

[1] Zandalinas S.I., Peláez-Vico M.Á., Sinha R., Pascual L.S., Mittler R. (2024). The impact of multifactorial stress combination on plants, crops, and ecosystems: How should we prepare for what comes next?. Plant J..

[2] Bibi F., Rahman A. (2023). An overview of climate change impacts on agriculture and their mitigation strategies. Agriculture.

[3] Vaštag E., Orlović S., Bojović M., Kesić L., Pap P., Stojnić S. (2022). The influence of powdery mildew on chlorophyll a fluorescence and stomatal characteristics of pedunculate oak (*Quercus robur* L.). Topola.

[4] Kesić L., Kovačević B., Milović M., Poljaković-Pajnik L., Pekeč S., Višacki V., Orlović S. (2024). Physiological responses of poplar and willow clones grown in pot trials on soil from landfills. Topola.

[5] Chen F., Chen L., Yan Z., Xu J., Feng L., He N., Guo M., Zhao J., Chen Z., Chen H. (2024). Recent advances of CRISPR-based genome editing for enhancing staple crops. Front. Plant Sci..

[6] Zhan X., Zhang F., Li N., Xu K., Wang X., Gao S., Yin Y., Yuan W., Chen W., Ren Z. (2024). CRISPR/Cas: An emerging toolbox for engineering virus resistance in plants. Plants.

[7] de la Fuente Tagarro C., Martín-González D., De Lucas A., Bordel S., Santos-Beneit F. (2024). Current knowledge on CRISPR strategies against antimicrobial-resistant bacteria. Antibiotics.

[8] Zhang X., Huang Z., Zhang Y., Wang W., Ye Z., Liang P., Sun K., Kang W., Tang Q., Yu X. (2024). Mitigating antibiotic resistance: The utilization of CRISPR technology in detection. Biosensors.

[9] Han W., She Q. (2017). CRISPR history: Discovery, characterization, and prosperity. Prog. Mol. Biol. Transl. Sci..

[10] Demirer G.S., Zhang H., Matos J.L., Goh N.S., Cunningham F.J., Sung Y., Chang R., Aditham A.J., Chio L., Cho M.J. (2019). High aspect ratio nanomaterials enable delivery of functional genetic material without DNA integration in mature plants. Nat. Nanotechnol..

[11] Kwak S.Y., Lew T.T.S., Sweeney C.J., Koman V.B., Wong M.H., Bohmert-Tatarev K., Snell K.D., Seo J.S., Chua N.H., Strano M.S. (2019). Chloroplast-selective gene delivery and expression in planta using chitosan-complexed single-walled carbon nanotube carriers. Nat. Nanotechnol..

[12] Lai C.M., Xiao X.S., Liu L.W., Lin X.D., Dou D.L., Cai H.Y., Mei Z.F., Yang F., Cheng Y., Qin Y. (2025). Nanotechnology Strategies in Plant Genetic Engineering: Intelligent Delivery and Precision Editing. Plants.

[13] Shivashakarappa K., Marriboina S., Dumenyo K., Taheri A., Yadegari Z. (2025). Nanoparticle-mediated gene delivery techniques in plant systems. Front. Nanotechnol..

[14] Makarova K.S., Wolf Y.I., Alkhnbashi O.S., Costa F., Shah S.A., Saunders S.J., Barrangou R., Brouns S.J., Charpentier E., Haft D.H. (2015). An updated evolutionary classification of CRISPR–Cas systems. Nat. Rev. Microbiol..

[15] Ma X. (2024). CRISPR Development and Application. Highlights Sci. Eng. Technol..

[16] Barrangou R., Marraffini L.A. (2014). CRISPR-Cas systems: Prokaryotes upgrade to adaptive immunity. Mol. Cell.

[17] Lee H., Sashital D.G. (2022). Creating memories: Molecular mechanisms of CRISPR adaptation. Trends Biochem. Sci..

[18] Nidhi S., Anand U., Oleksak P., Tripathi P., Lal J.A., Thomas G., Kuca K., Tripathi V. (2021). Novel CRISPR–Cas systems: An updated review of the current achievements, applications, and future research perspectives. Int. J. Mol. Sci..

[19] Zakrzewska M., Burmistrz M. (2023). Mechanisms regulating the CRISPR-Cas systems. Front. Microbiol..

[20] Mohamadi S., Bostanabad S.Z., Mirnejad R. (2020). CRISPR arrays: A review on its mechanism. J. Appl. Biotechnol. Rep..

[21] Laforest L.C., Nadakuduti S.S. (2022). Advances in delivery mechanisms of CRISPR gene-editing reagents in plants. Front. Genome Ed..

[22] Ajdanian L., Villot S., Karikari B., Torkamaneh D. (2026). Technological advances in trait development: From conventional breeding and untargeted mutagenesis to precision genome editing. Genome.

[23] Bevan M., Barnes W.M., Chilton M.D. (1983). Strcuture and transcription of the nopaline synthase gene region of T-DNA. Nucleic Acids Res..

[24] Kocsisova Z., Coneva V. (2023). Strategies for delivery of CRISPR/Cas-mediated genome editing to obtain edited plants directly without transgene integration. Front. Genome Ed..

[25] Ozyigit I.I., Yucebilgili Kurtoglu K. (2020). Particle bombardment technology and its applications in plants. Mol. Biol. Rep..

[26] La Lacroix B., Citovsky V., Rustgi S., Luo H. (2020). Biolistic Approach for Transient Gene Expression Studies in Plants.

[27] Tripathi A., Shukla S. (2024). Methods of genetic transformation: Major emphasis to crop plants. J. Microbiol. Biotechnol. Food Sci..

[28] Galovic V. (2010). Mature embryo-derived wheat transformation with major stress-modulated antioxidant target gene. Arch. Biol. Sci..

[29] Sota V., Jevremović S., Abraham E., Daničić V., Bošnjak D., Nacheva L., Cvjetković B., Andonovski V., Bogunović S., Kongjika E. (2025). The Balkan Region and the “Nano Gap”: An Underexplored Dimension of In Vitro Biotechnology for Woody Plants. Plants.

[30] Wu S., Zhu H., Liu J., Yang Q., Shao X., Bi F., Hu C., Huo H., Chen K., Yi G. (2020). Establishment of a PEG-mediated protoplast transformation system based on DNA and CRISPR/Cas9 ribonucleoprotein complexes for banana. BMC Plant Biol..

[31] Wu M., Chen A., Li X., Li X., Hou X., Liu X. (2024). Advancements in delivery strategies and non-tissue culture regeneration systems for plant genetic transformation. Adv. Biotechnol..

[32] Batool S., Li Z., Zhang D., Shi P., Htwe Y.M., Nie H., Ma M., Su H., Fang X., Ahmed M.A. (2025). PEG-mediated transformation and CRISPR/Cas9 gene editing of CnPDS in coconut protoplast. Ind. Crops Prod..

[33] Mehrotra S., Goyal V. (2012). Agrobacterium-mediated gene transfer in plants and biosafety considerations. Appl. Biochem. Biotechnol..

[34] Hwang H.H., Yu M., Lai E.M. (2017). Agrobacterium-mediated plant transformation: Biology and applications. Arab. Book.

[35] Rustgi S., Naveed S., Windham J., Zhang H., Demirer G.S. (2022). Plant biomacromolecule delivery methods in the 21st century. Front. Genome Ed..

[36] Javaid A., Hameed S., Li L., Zhang Z., Zhang B., Rahman M.U. (2024). Can nanotechnology and genomics innovations trigger agricultural revolution and sustainable development?. Funct. Integr. Genom..

[37] Ghosh P., Goswami A., Ratnaparkhi P., Goswami A., Polikarpov I., Sil M. (2026). Evolution of plant gene delivery: From biolistic to next-generation nanocarriers. Plant Gene.

[38] Saglam M., Tsakirpaloglou N., Bridgeland A., Miller R., Thomson M.J., Septiningsih E.M. (2026). Carbon nanotube and carbon dot mediated plasmid DNA delivery in cowpea leaves. PLoS ONE.

[39] Walther J., Porenta D., Wilbie D., Seinen C., Benne N., Yang Q., de Jong O.G., Lei Z., Mastrobattista E. (2024). Comparative analysis of lipid Nanoparticle-Mediated delivery of CRISPR-Cas9 RNP versus mRNA/sgRNA for gene editing in vitro and in vivo. Eur. J. Pharm. Biopharm..

[40] Oliva R., Ji C., Atienza-Grande G., Huguet-Tapia J.C., Perez-Quintero A., Li T., Eom J.S., Li C., Nguyen H., Liu B. (2019). Broad-spectrum resistance to bacterial blight in rice using genome editing. Nat. Biotechnol..

[41] Nekrasov V., Wang C., Win J., Lanz C., Weigel D., Kamoun S. (2017). Rapid generation of a transgene-free powdery mildew resistant tomato by genome deletion. Sci. Rep..

[42] Shi J., Gao H., Wang H., Lafitte H.R., Archibald R.L., Yang M., Hakimi S.M., Mo H., Habben J.E. (2017). ARGOS 8 variants generated by CRISPR-Cas9 improve maize grain yield under field drought stress conditions. Plant Biotechnol. J..

[43] Do P.T., Nguyen C.X., Bui H.T., Tran L.T., Stacey G., Gillman J.D., Zhang Z.J., Stacey M.G. (2019). Demonstration of highly efficient dual gRNA CRISPR/Cas9 editing of the homeologous GmFAD2–1A and GmFAD2–1B genes to yield a high oleic, low linoleic and α-linolenic acid phenotype in soybean. BMC Plant Biol..

[44] Zhou X., Jacobs T.B., Xue L.J., Harding S.A., Tsai C.J. (2015). Exploiting SNPs for biallelic CRISPR mutations in the outcrossing woody perennial Populus reveals 4-coumarate: CoA ligase specificity and redundancy. New Phytol..

[45] Dai Y., Hu G., Dupas A., Medina L., Blandels N., San Clemente H., Ladouce N., Badawi M., Hernandez-Raquet G., Mounet F. (2020). Implementing the CRISPR/Cas9 technology in Eucalyptus hairy roots using wood-related genes. Int. J. Mol. Sci..

[46] Endo A., Masafumi M., Kaya H., Toki S. (2016). Efficient targeted mutagenesis of rice and tobacco genomes using Cpf1 from *Francisella novicida*. Sci. Rep..

[47] Zhang Y., Cheng Y., Fang H., Roberts N., Zhang L., Vakulskas C.A., Niedz R.P., Culver J.N., Qi Y. (2022). Highly efficient genome editing in plant protoplasts by ribonucleoprotein delivery of CRISPR-Cas12a nucleases. Front. Genome Ed..

[48] Kim H., Kim S.T., Ryu J., Kang B.C., Kim J.S., Kim S.G. (2017). CRISPR/Cpf1-mediated DNA-free plant genome editing. Nat. Commun..

[49] Bernabé-Orts J.M., Casas-Rodrigo I., Minguet E.G., Landolfi V., Garcia-Carpintero V., Gianoglio S., Vázquez-Vilar M., Granell A., Orzaez D. (2019). Assessment of Cas12a-mediated gene editing efficiency in plants. Plant Biotechnol. J..

[50] An Y., Geng Y., Yao J., Fu C., Lu M., Wang C., Du J. (2020). Efficient genome editing in Populus using CRISPR/Cas12a. Front. Plant Sci..

[51] Zong Y., Wang Y., Li C., Zhang R., Chen K., Ran Y., Qiu J.L., Wang D., Gao C. (2017). Precise base editing in rice, wheat and maize with a Cas9-cytidine deaminase fusion. Nat. Biotechnol..

[52] Yang B., Yang L., Chen J. (2019). Development and application of base editors. CRISPR J..

[53] Zhang R., Zheng Z., Li G., Zheng X., Su L., Yuan X., Li T., Tan J., Zeng D., Zhang S. (2025). Plant base editing: A decade of progress and future applications. aBIOTECH.

[54] Veillet F., Perrot L., Chauvin L., Kermarrec M.P., Guyon-Debast A., Chauvin J.E., Nogué F., Mazier M. (2019). Transgene-free genome editing in tomato and potato plants using agrobacterium-mediated delivery of a CRISPR/Cas9 cytidine base editor. Int. J. Mol. Sci..

[55] Kang B.C., Yun J.Y., Kim S.T., Shin Y., Ryu J., Choi M., Woo J.W., Kim J.S. (2018). Precision genome engineering through adenine base editing in plants. Nat. Plants.

[56] Yao T., Yuan G., Lu H., Liu Y., Zhang J., Tuskan G.A., Muchero W., Chen J.G., Yang X. (2023). CRISPR/Cas9-based gene activation and base editing in Populus. Hortic. Res..

[57] Rocha D.C., Omoregbee M.O., Contiliani D.F., Mandlik R., Li G., Mascoveto J., Coleman G., Culver J.N., Leal D.R., de Souza A.A. (2025). Transgene-free genome editing in citrus and poplar trees using positive and negative selection markers. Plant Cell Rep..

[58] Vats S., Kumar J., Sonah H., Zhang F., Deshmukh R. (2024). Prime editing in plants: Prospects and challenges. J. Exp. Bot..

[59] Chen P.J., Liu D.R. (2023). Prime editing for precise and highly versatile genome manipulation. Nat. Rev. Gen..

[60] Xu R., Liu X., Li J., Qin R., Wei P. (2021). Identification of herbicide resistance OsACC1 mutations via in planta prime-editing-library screening in rice. Nat. Plants.

[61] Jiang Y.Y., Chai Y.P., Lu M.H., Han X.L., Lin Q., Zhang Y., Zhang Q., Zhou Y., Wang X.C., Gao C. (2020). Prime editing efficiently generates W542L and S621I double mutations in two ALS genes in maize. Genome Biol..

[62] Lin Q., Zong Y., Xue C., Wang S., Jin S., Zhu Z., Wang Y., Anzalone A.V., Raguram A., Doman J.L. (2020). Prime genome editing in rice and wheat. Nat. Biotechnol..

[63] Biswas S., Bridgeland A., Irum S., Thomson M.J., Septiningsih E.M. (2022). Optimization of prime editing in rice, peanut, chickpea, and cowpea protoplasts by restoration of GFP activity. Int. J. Mol. Sci..

[64] Lu Y., Tian Y., Shen R., Yao Q., Zhong D., Zhang X., Zhu J.K. (2020). Precise genome modification in tomato using an improved prime editing system. Plant Biotechnol. J..

[65] Zou J., Li Y., Wang K., Wang C., Zhuo R. (2024). Prime editing enables precise genome modification of a Populus hybrid. aBIOTECH.

[66] Li J., Zhang C., He Y., Li S., Yan L., Li Y., Zhu Z., Xia L. (2023). Plant base editing and prime editing: The current status and future perspectives. J. Integr. Plant Biol..

[67] Cunningham F.J., Goh N.S., Demirer G.S., Matos J.L., Landry M.P. (2018). Nanoparticle-mediated delivery towards advancing plant genetic engineering. Trends Biotechnol..

[68] Bélanger J.G., Copley T.R., Hoyos-Villegas V., Charron J.B., O’Donoughue L. (2024). A comprehensive review of in planta stable transformation strategies. Plant Methods.

[69] Maharjan B.K., Islam M.T., Muzaffar A., Tschaplinski T.J., Tuskan G.A., Chen J.G., Yang X. (2025). Woody Plant Transformation: Current Status, Challenges, and Future Perspectives. Plants.

[70] Li W., Lin Y.C.J., Chen Y.L., Zhou C., Li S., De Ridder N., Oliveira D.M., Zhang L., Zhang B., Wang J.P. (2024). Woody plant cell walls: Fundamentals and utilization. Mol. Plant.

[71] Altpeter F., Springer N.M., Bartley L.E., Blechl A.E., Brutnell T.P., Citovsky V., Conrad L.J., Gelvin S.B., Jackson D.P., Kausch A.P. (2016). Advancing crop transformation in the era of genome editing. Plant Cell.

[72] Jia Y., Yao X., Zhao M., Zhao Q., Du Y., Yu C., Xie F. (2015). Comparison of soybean transformation efficiency and plant factors affecting transformation during the Agrobacterium infection process. Int. J. Mol. Sci..

[73] da Silva R.G., Coppede J.S., Silva J.O., Zingaretti S.M. (2018). Efficient method for Agrobacterium-mediated genetic transformation of tobacco nodal segments. Genet. Mol. Res..

[74] Wang X., Chen S., Zhang H., Luo P., Zhou F., Zeng B., Xu J., Fan C. (2023). Agrobacterium-mediated genetic transformation of the most widely cultivated superior clone *Eucalyptus urophylla* × *E. grandis* DH32-29 in Southern China. Front. Plant Sci..

[75] Wen S.S., Ge X.L., Wang R., Yang H.F., Bai Y.E., Guo Y.H., Zhang J., Lu M.Z., Zhao S.T., Wang L.Q. (2022). An efficient Agrobacterium-mediated transformation method for hybrid poplar 84K (*Populus alba* × *P. glandulosa*) using calli as explants. Int. J. Mol. Sci..

[76] Bekalu Z.E., Panting M., Bæksted Holme I., Brinch-Pedersen H. (2023). Opportunities and challenges of in vitro tissue culture systems in the era of crop genome editing. Int. J. Mol. Sci..

[77] Ikeuchi M., Sugimoto K., Iwase A. (2013). Plant callus: Mechanisms of induction and repression. Plant Cell.

[78] Lee K., Wang K. (2023). Strategies for genotype-flexible plant transformation. Curr. Opin. Biotechnol..

[79] Hajiahmadi Z., Movahedi A., Wei H., Li D., Orooji Y., Ruan H., Zhuge Q. (2019). Strategies to increase on-target and reduce off-target effects of the CRISPR/Cas9 system in plants. Int. J. Mol. Sci..

[80] Wang Y., Zafar N., Ali Q., Manghwar H., Wang G., Yu L., Ding X., Ding F., Hong N., Wang G. (2022). CRISPR/Cas genome editing technologies for plant improvement against biotic and abiotic stresses: Advances, limitations, and future perspectives. Cells.

[81] Manghwar H., Li B., Ding X., Hussain A., Lindsey K., Zhang X., Jin S. (2020). CRISPR/Cas systems in genome editing: Methodologies and tools for sgRNA design, off-target evaluation, and strategies to mitigate off-target effects. Adv. Sci..

[82] Li A., Zafar M.M., Farooq Z., Ahmed S.R., Ijaz A., Anwar Z., Abbas H., Tariq M.S., Tariq H., Mustafa M. (2023). Breakthrough in CRISPR/Cas system: Current and future directions and challenges. Biotechnol. J..

[83] Biswas S., Tian J., Li R., Chen X., Luo Z., Chen M., Zhao X., Zhang D., Persson S., Yuan Z. (2020). Investigation of CRISPR/Cas9-induced SD1 rice mutants highlights the importance of molecular characterization in plant molecular breeding. J. Genet. Genom..

[84] Wang X., Tu M., Wang Y., Yin W., Zhang Y., Wu H., Gu Y., Li Z., Xi Z., Wang X. (2021). Whole-genome sequencing reveals rare off-target mutations in CRISPR/Cas9-edited grapevine. Hortic. Res..

[85] Zhang Q., Xing H.L., Wang Z.P., Zhang H.Y., Yang F., Wang X.C., Chen Q.J. (2018). Potential high-frequency off-target mutagenesis induced by CRISPR/Cas9 in Arabidopsis and its prevention. Plant Mol. Biol..

[86] Goralogia G.S., Andreatta I.M., Conrad V., Xiong Q., Vining K.J., Strauss S.H. (2024). Rare but diverse off-target and somatic mutations found in field and greenhouse grown trees expressing CRISPR/Cas9. Front. Bioeng. Biotechnol..

[87] Rabiee N. (2023). Natural components as surface engineering agents for CRISPR delivery. Environ. Res..

[88] Schaming D., Remita H. (2015). Nanotechnology: From the ancient time to nowadays. Found. Chem..

[89] Wagay O.A., Khan S., Rafeeq J., Pala N.A., Bhat G.M., Dutt V., Peerzada I.A., Malik A.R., Sofi P.A., Mushtaq T. (2023). Nanotechnology and its potential application in forest and forest-based industries: A review. SKUAST J. Res..

[90] Kah M., Kookana R.S., Gogos A., Bucheli T.D. (2018). A critical evaluation of nanopesticides and nanofertilizers against their conventional analogues. Nat. Nanotechnol..

[91] Demirer G.S., Zhang H., Goh N.S., González-Grandío E., Landry M.P. (2019). Carbon nanotube–mediated DNA delivery without transgene integration in intact plants. Nat. Protoc..

[92] Santos P.A., Biraku X., Nielsen E., Ozketen A.C., Ozketen A.A., Hakki E.E. (2025). Agricultural nanotechnology for a safe and sustainable future: Current status, challenges, and beyond. J. Sci. Food Agric..

[93] Wei X., Li S., Wang W., Zhang X., Zhou W., Xie S., Liu H. (2022). Recent advances in structure separation of single-wall carbon nanotubes and their application in optics, electronics, and optoelectronics. Adv. Sci..

[94] Wang T., Li J., Hu R., Shentu X., Ye Z., Yu X., Sun K. (2025). Nanoparticle-Mediated Nucleic Acid Delivery Systems in Plant Biotechnology: Recent Advances and Emerging Challenges. Plants.

[95] Syduzzaman M., Saad M.S.I., Piam M.F., Talukdar T.A., Shobdo T.T., Pritha N.M. (2025). Carbon nanotubes: Structure, properties and applications in the aerospace industry. Results Mater..

[96] Ali Z., Serag M.F., Demirer G.S., Torre B., Di Fabrizio E., Landry M.P., Habuchi S., Mahfouz M. (2022). DNA–Carbon nanotube binding mode determines the efficiency of carbon nanotube-mediated DNA delivery to intact plants. ACS Appl. Nano Mater..

[97] Taha E.A., Lee J., Hotta A. (2022). Delivery of CRISPR-Cas tools for in vivo genome editing therapy: Trends and challenges. J. Control. Release.

[98] Kim M., Hwang Y., Lim S., Jang H.K., Kim H.O. (2024). Advances in nanoparticles as non-viral vectors for efficient delivery of CRISPR/Cas9. Pharmaceutics.

[99] Farsani A.M., Mokhtari N., Nooraei S., Bahrulolum H., Akbari A., Farsani Z.M., Khatami S., sadat Ebadi M., Ahmadian G. (2024). Lipid nanoparticles: The game-changer in CRISPR-Cas9 genome editing. Heliyon.

[100] Kazemian P., Yu S.Y., Thomson S.B., Birkenshaw A., Leavitt B.R., Ross C.J. (2022). Lipid-nanoparticle-based delivery of CRISPR/Cas9 genome-editing components. Mol. Pharma..

[101] Panda P., Mohapatra R. (2025). Advancements in DNA nanotechnology for targeted drug delivery: Design strategies and applications. Hybrid Adv..

[102] Bharti N., Modi U., Bhatia D., Solanki R. (2026). Engineering delivery platforms for CRISPR-Cas and their applications in healthcare, agriculture and beyond. Nanoscale Adv..

[103] Huang Y., Chen Z., Huang H., Ding S., Zhang M. (2025). Important applications of DNA nanotechnology combined with CRISPR/Cas systems in biotechnology. RSC Adv..

[104] Sun W., Ji W., Hall J.M., Hu Q., Wang C., Beisel C.L., Gu Z. (2015). Self-assembled DNA nanoclews for the efficient delivery of CRISPR–Cas9 for genome editing. Angew. Chem..

[105] Zhang H., Demirer G.S., Zhang H., Ye T., Goh N.S., Aditham A.J., Cunningham F.J., Fan C., Landry M.P. (2019). DNA nanostructures coordinate gene silencing in mature plants. Proc. Natl. Acad. Sci. USA.

[106] Leal G.G., dos Santos Y.B.L., Ito M.E.Q.S., de Souza W.R. (2025). Big things come in small packages: Using nanomaterials for plant genetic engineering. Plant Nano Biol..

[107] Fanarraga M.L., Hevia L.G. (2026). Silica nanoparticles as advanced platforms for nucleic acid delivery. Mater. Today Bio..

[108] Xu X., Koivisto O., Liu C., Zhou J., Miihkinen M., Jacquemet G., Wang D., Rosenholm J.M., Shu Y., Zhang H. (2021). Effective delivery of the CRISPR/Cas9 system enabled by functionalized mesoporous silica nanoparticles for GFP-tagged paxillin knock-in. Adv. Ther..

[109] Sandhya D., Jogam P., Allini V.R., Abbagani S., Alok A. (2020). The present and potential future methods for delivering CRISPR/Cas9 components in plants. J. Genet. Eng. Biotechnol..

[110] George A.C.S. (2018). Magnetofection of Tobacco Protoplasts: A Novel Mechanism for Plant Transformation. Doctoral Dissertation.

[111] Zhao X., Meng Z., Wang Y., Chen W., Sun C., Cui B., Cui J., Yu M., Zeng Z., Guo S. (2017). Pollen magnetofection for genetic modification with magnetic nanoparticles as gene carriers. Nat. Plants.

[112] Farooq N., Ather L., Shafiq M., Nawaz-ul-Rehman M.S., Haseeb M., Anjum T., Abbas Q., Hussain M., Ali N., Asad Abbas S.A.A. (2022). Magnetofection approach for the transformation of okra using green iron nanoparticles. Sci. Rep..

[113] Park C.W., Choi J.Y., Son Y.J., Kim D.H., Li H., Liang W., Lee C., Jung K.H., Kim Y.J. (2024). Magnetofected pollen gene delivery system could generate genetically modified Cucumis sativus. Hortic. Res..

[114] Vejlupkova Z., Warman C., Sharma R., Scheller H.V., Mortimer J.C., Fowler J.E. (2020). No evidence for transient transformation via pollen magnetofection in several monocot species. Nat. Plants.

[115] Zhang M., Ma X., Jin G., Han D., Xue J., Du Y., Chen X., Yang F., Zhao C., Zhang X. (2023). A modified method for transient transformation via pollen magnetofection in Lilium germplasm. Int. J. Mol. Sci..

[116] Lv Z., Jiang R., Chen J., Chen W. (2020). Nanoparticle-mediated gene transformation strategies for plant genetic engineering. Plant J..

[117] Liu S., Zheng Y., Pan L., Wang W., Li Y., Liu Z., Zhang X. (2025). Nanodelivery of nucleic acids for plant genetic engineering. Discov. Nano.

[118] YingYing J., Balasubramanian B., Park S., Anand A., Meyyazhagan A., Pappusamy M., Paari K.A., Kamyab H., Chelliapan S. (2025). Green nanoparticles in agriculture: Enhancing crop growth and stress tolerance. Plant Stress.

[119] Karayianni M., Sentoukas T., Skandalis A., Pippa N., Pispas S. (2023). Chitosan-based nanoparticles for nucleic acid delivery: Technological aspects, applications, and future perspectives. Pharmaceutics.

[120] Kim N.O.T., Parushi N., Po-Ting C., Chwen-Jen S., Yung-Chuan L., Chia-Hung K. (2025). Advances in chitosanase research: From structure and function to green biocatalytic production of chitooligosaccharides. Catalysts.

[121] Kani A.K., Khan Z., Sena S., Akhtar N., Alreshdi M.A., Yadav K.K., Alkahtani A.M., Wani A.W., Rahayu F., Tafakresnanto C. (2024). Carbon nanotubes in plant dynamics: Unravelling multifaceted roles and phytotoxic implications. Plant Physiol. Biochem..

[122] Ahmad Z., Niyazi S., Firdoos A., Wang C., Manzoor M.A., Ramakrishnan M., Upadhyay A., Ding Y. (2024). Enhancing plant resilience: Nanotech solutions for sustainable agriculture. Heliyon.

[123] Jat S.K., Bhattacharya J., Sharma M.K. (2020). Nanomaterial based gene delivery: A promising method for plant genome engineering. J. Mater. Chem. B.

[124] Möller K., Müller K., Engelke H., Bräuchle C., Wagner E., Bein T. (2016). Highly efficient siRNA delivery from core–shell mesoporous silica nanoparticles with multifunctional polymer caps. Nanoscale.

[125] Hu P., An J., Faulkner M.M., Wu H., Li Z., Tian X., Giraldo J.P. (2020). Nanoparticle charge and size control foliar delivery efficiency to plant cells and organelles. ACS Nano.

[126] Galović V., Joseph M.P., Pekeč S., Vasić V., Vasić S., Szabados L. (2020). Characterization of abiotic stress-responsive RD29B and RD17 genes in different poplar clones. Topola.

[127] Zhou Y., Xu S., Jiang N., Zhao X., Bai Z., Liu J., Yao W., Tang Q., Xiao G., Lv C. (2022). Engineering of rice varieties with enhanced resistances to both blast and bacterial blight diseases via CRISPR/Cas9. Plant Biotechnol. J..

[128] Yuan X., Xu K., Yan F., Liu Z., Spetz C., Zhou H., Wang X., Jin H., Wang X., Liu Y. (2024). CRISPR/Cas9-mediated resistance to wheat dwarf virus in hexaploid wheat (*Triticum aestivum* L.). Viruses.

[129] Ito Y., Nishizawa-Yokoi A., Endo M., Mikami M., Toki S. (2015). CRISPR/Cas9-mediated mutagenesis of the RIN locus that regulates tomato fruit ripening. Biochem. Biophys. Res. Commun..

[130] González M.N., Massa G.A., Andersson M., Turesson H., Olsson N., Fält A.S., Storani L., Décima Oneto C.A., Hofvander P., Feingold S.E. (2020). Reduced enzymatic browning in potato tubers by specific editing of a polyphenol oxidase gene via ribonucleoprotein complexes delivery of the CRISPR/Cas9 system. Front. Plant Sci..

[131] Torney F., Trewyn B.G., Lin V.S.Y., Wang K. (2007). Mesoporous silica nanoparticles deliver DNA and chemicals into plants. Nat. Nanotechnol..

[132] Chang F.P., Kuang L.Y., Huang C.A., Jane W.N., Hung Y., Hsing Y.I.C., Mou C.Y. (2013). A simple plant gene delivery system using mesoporous silica nanoparticles as carriers. J. Mater. Chem. B.

[133] Demirer G.S., Zhang H., Goh N.S., Pinals R.L., Chang R., Landry M.P. (2020). Carbon nanocarriers deliver siRNA to intact plant cells for efficient gene knockdown. Sci. Adv..

[134] Zhang H., Zhang H., Demirer G.S., González-Grandío E., Fan C., Landry M.P. (2020). Engineering DNA nanostructures for siRNA delivery in plants. Nat. Protoc..

[135] Martin-Ortigosa S., Peterson D.J., Valenstein J.S., Lin V.S.Y., Trewyn B.G., Lyznik L.A., Wang K. (2014). Mesoporous silica nanoparticle-mediated intracellular Cre protein delivery for maize genome editing via loxP site excision. Plant Physiol..

[136] Tawfik E., Ahmed M.F. (2022). Chitosan nanoparticles as a new technique in gene transformation into different plants tissues. Nat. Resour. Hum. Health.

[137] Abdel-Razik A.B., Hammad I.A., Tawfik E. (2017). Transformation of thionin genes using chitosan nanoparticle into potato plant to be resistant to fungal infection. IOSR J. Biotechnol. Biochem..

[138] Allah K.W.A., Alabasey E.E.D.G.H., Ahmed K.Z., Hussien E.T., Razik A.B.A. (2023). *Phoenix dactylifera* in vitro culture and transformation of Thio-60 antifungal gene via chitosan nanoparticle. Plant Cell Tiss. Organ Cult..

[139] Mundorf J., Simon S., Engelhard M. (2025). The European Commission’s regulatory proposal on new genomic techniques in plants: A focus on equivalence, complexity, and artificial intelligence. Environ. Sci. Eur..

[140] Thakur S., Kaur S., Adhikari S., Sabharwal P., Fu Y., Meru G. (2025). Turning susceptibility into strength: A new era of durable resistance in plants through genome editing. Plants.

[141] Fernández Ríos D., Quintana S.A., Gómez Paniagua P., Arrúa A.A., Brozón G.R., Bertoni Hicar M.S., Castro Alegría A., Goberna M.F. (2025). Regulatory challenges and global trade implications of genome editing in agriculture. Front. Bioeng. Biotechnol..

[142] Custers R., Dima O. (2022). “Genome-edited crops and 21st century food system challenges” depth analysis. Panel Future Sci. Technol..

[143] Waltz E. (2022). GABA-enriched tomato is first CRISPR-edited food to enter market. Nat. Biotechnol..

[144] Bruegmann T., Fendel A., Zahn V., Fladung M., Ricroch A., Eriksson D., Miladinović D., Sweet J., Van Laere K., Woźniak-Gientka E. (2024). Genome Editing in Forest Trees. A Roadmap for Plant Genome Editing.

[145] Ghosh M., Kumar R., Prasad M., Kumar R., Ghosh M., Syed S.M., Chakravarti S. (2024). Regulatory Issues in Nanotechnology. Nanotechnology Theranostics in Livestock Diseases and Management.

[146] Xin X., Judy J.D., Sumerlin B.B., He Z. (2020). Nano-enabled agriculture: From nanoparticles to smart nanodelivery systems. Environ. Chem..

[147] Naeem M., Hoque M.Z., Ovais M., Basheer C., Ahmad I. (2021). Stimulus-responsive smart nanoparticles-based CRISPR-Cas delivery for therapeutic genome editing. Int. J. Mol. Sci..

[148] Kim M.G., Go M.J., Kang S.H., Jeong S.H., Lim K. (2025). Revolutionizing CRISPR technology with artificial intelligence. Exp. Mol. Med..

[149] Du J., Wu Q., Liu C., Wang N., Gong C. (2025). Crispr delivery systems for organ-specific targeting: Advances and challenges. Precis. Med. Eng..

[150] Yadav A.R., Upare M.M., Desai S.R., Sachdeo R.A., Mahamuni S.S., Shah N.V. (2026). Nanotechnology Based CRISPR Delivery Platforms in Therapeutic Genome Engineering. Int. J. Drug Deliv. Technol..

